# Focal Neuroendocrine Differentiation of Conventional Prostate Adenocarcinoma as a Prognostic Factor after Radical Prostatectomy: A Systematic Review and Meta-Analysis

**DOI:** 10.3390/ijms20061374

**Published:** 2019-03-19

**Authors:** Mehdi Kardoust Parizi, Takehiro Iwata, Shoji Kimura, Florian Janisch, Mohammad Abufaraj, Pierre I. Karakiewicz, Dmitry Enikeev, Leonid M. Rapoport, Georg Hutterer, Shahrokh F. Shariat

**Affiliations:** 1 Department of Urology, Medical University of Vienna, A-1090 Vienna, Austria; m.kardoust@yahoo.com (M.K.P.); takehiroiwata1221@gmail.com (T.I.); shoji.kimura-1228@hotmail.co.jp (S.K.); drfjanisch@gmail.com (F.J.); dr.abufaraj@gmail.com (M.A.); 2 Department of Urology, Shariati Hospital, Tehran University of Medical Sciences, Teheran 1411713135, Iran; 3 Department of Urology, Okayama University Graduate School of Medicine, Dentistry and Pharmaceutical Sciences, Okayama 700-8558, Japan; 4 Department of Urology, Jikei University School of Medicine, Tokyo 105-8461, Japan; 5 Department of Urology, University Medical Center Hamburg-Eppendorf, 20246 Hamburg, Germany; 6 Department of Special Surgery, Jordan University Hospital, The University of Jordan, Amman 11942, Jordan; 7 Cancer Prognostics and Health Outcomes Unit, University of Montreal Health Center, Montreal, QC H3h 1s8, Canada; pierre.karakiewicz@umontreal.ca; 8 Centre de recherche du Centre Hospitalier de l’Université de Montréal (CR-CHUM), Institut du Cancer de Montréal, Montréal, QC H3h 1s8, Canada; 9 Institute for Urology and Reproductive Health, Sechenov University, Moscow 119991, Russia; dvenikeev@gmail.com (D.E.); urologystatement1@yandex.com (L.M.R.); 10 Department of Urology, Medical University Graz, A-8036 Graz, Austria; g.hutterer@gmail.com; 11 Department of Urology, Weill Cornell Medical College, New York, NY 10011, USA; 12 Department of Urology, University of Texas Southwestern Medical Center, Dallas, TX 75390, USA; 13 Karl Landsteiner Institute of Urology and Andrology, A-1090 Vienna, Austria

**Keywords:** neuroendocrine differentiation, chromogranin A, prostate cancer, radical prostatectomy, oncological outcome, survival

## Abstract

The biologic and prognostic value of focal neuroendocrine differentiation (NED) in conventional prostate adenocarcinoma (PC) patients who undergo radical prostatectomy (RP) remains controversial. In this systematic review and meta-analysis, we assessed the association of focal NED in conventional PC with oncological outcomes after RP. A literature search using PubMed, Scopus, Web of Science, and Cochrane Library was conducted on December 2018 to find relevant studies according to the Preferred Reporting Items for Systematic Review and Meta-Analysis (PRISMA) guidelines. We used a fixed-effect model to analyze the impact of focal NED in RP specimen on progression-free survival defined by biochemical recurrence (BCR). A total of 16 studies with the outcomes of disease progression and survival were eligible. No patient in these studies received androgen deprivation therapy prior to RP. Eleven studies found no significant correlation between focal NED and outcomes of interest, while five studies reported a significant association of focal NED assessed by immunohistochemical chromogranin A or serotonin staining with BCR or survival. Focal NED was associated with higher BCR rates after RP with a pooled HR of 1.39 (95% CI 1.07‒1.81) in five studies. No heterogeneity was reported in this analysis (I^2^ = 21.7%, *p* = 0.276). In conclusion, focal NED in conventional PC is associated with worse prognosis after RP. Its presence should be reported in pathologic reports and its true clinical impact should be assessed in well-designed prospective controlled studies.

## 1. Introduction

Prostate cancer (PC) is the most common solid cancer and the second most common cause of cancer-related death in men [1]. Over 90% of newly diagnosed PCs in developed countries are clinically localized to the origin. The standard treatment for these tumors is either active surveillance or local therapy with radical prostatectomy (RP) or radiation therapy. While these therapies result in durable local and distant disease control [2,3]. A significant number of patients eventually experience biochemical recurrence (BCR) despite effective definitive local therapy with curative intent (up to 35% at 10 years following RP) [4,5,6,7]. 

Recently, increasing attention has been given to neuroendocrine differentiation (NED) of PC recognizing its potential diagnostic, prognostic, and therapeutic utility [8]. Neuroendocrine cells are androgen-independent because of their negative androgen receptor expression [8]. NED is an important factor influencing the development of PC toward an androgen-independent, lethal phenotype.

Focal NED in conventional prostatic adenocarcinoma (PC) is one of the pathologically defined neuroendocrine manifestations in prostate gland, and its diagnosis is based on the detection of neuroendocrine cells using immunohistochemical analysis of biomarkers such as chromogranin A (CgA) and serotonin. Poorly differentiated prostatic neuroendocrine carcinomas including small and large cell carcinoma have been shown to harbor an aggressive clinical behavior and poor prognosis [9]. However, the prognostic value of focal NED in conventional PC remains controversial and its diagnostic and, specifically, clinical impact is poorly investigated.

To elucidate the prognostic value of focal NED, we performed a systematic review and meta-analysis investigating the impact of focal NED in conventional PC after RP on oncological outcomes including disease progression (i.e., BCR, local recurrence, and distant metastasis) and survival outcomes.

## 2. Results

### 2.1. Results of Search

**Figure 1:**
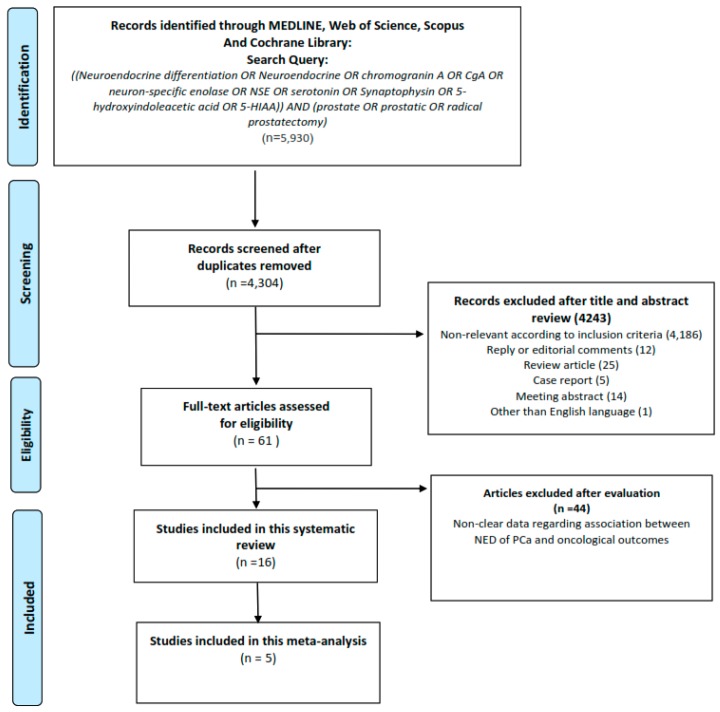
Preferred Reporting Items for Systematic Review and Meta-Analysis (PRISMA) flow chart for article selection process to analyze the impact of focal neuroendocrine differentiation in conventional prostate adenocarcinoma and oncological outcomes.

A total of 5930 studies were found for an initial assessment. Of these, 1626 duplicates were removed. After exclusion of non-relevant studies, review articles, meeting abstracts, case reports, replies, expert opinions, editorials or commentaries, and studies in languages other than English, 61 studies were reviewed. We finally identified 16 studies for systematic review and 5 studies for qualitative meta-analysis (Figure 1).

### 2.2. Characteristics of the Included Studies

The studies’ characteristics and patients’ clinical data are summarized in Table 1 and Table 2, respectively. The 16 studies comprised 2039 patients treated with RP. Included patients in these studies received no androgen deprivation therapy (ADT) prior to RP (as ADT can be a driver of neuroendocrine differentiation). The examined population was Northern American in seven studies, European in seven and Asian in two. All studies were designed retrospectively and were published between 1994 and 2017. All 16 included studies assessed CgA as a tissue marker for NED. Four [10,11,12,13] and three [10,14,15] studies used serotonin and neuron specific enolase (NSE) markers in addition to CgA to evaluate NED, respectively. BCR, the most frequently used oncologic outcome after RP, was reported in 13 studies [10,12,13,14,15,16,17,18,19,20,21,22,23]. Other reported oncologic endpoints included local recurrence, distant metastasis, and cancer-specific and overall survival. Follow-up ranged from 17.1 months to 17.3 years. Eleven studies found no significant association of focal NED with any oncologic outcome. Five studies comprising a total 1013 patients, in contrast, demonstrated a significant association of focal NED, as assessed using CgA or serotonin staining, with the prespecified oncologic endpoints of interest. [11,17,19,22,24]. The risk of bias in these16 studies is shown in Table 3.

### 2.3. Meta-Analysis

**Figure 2:**
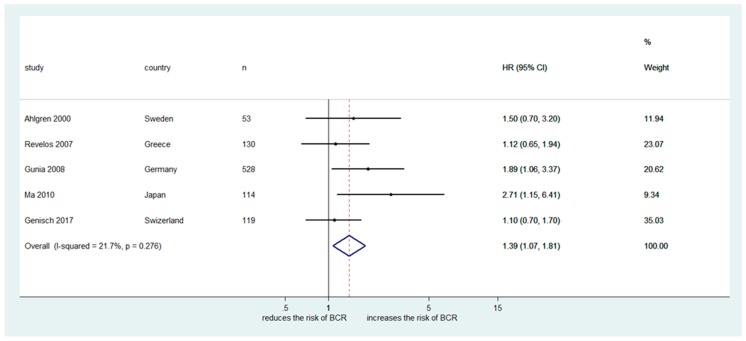
Forest plot presenting significant association of focal neuroendocrine differentiation in conventional prostate adenocarcinoma and biochemical recurrence after radical prostatectomy.

**Figure 3:**
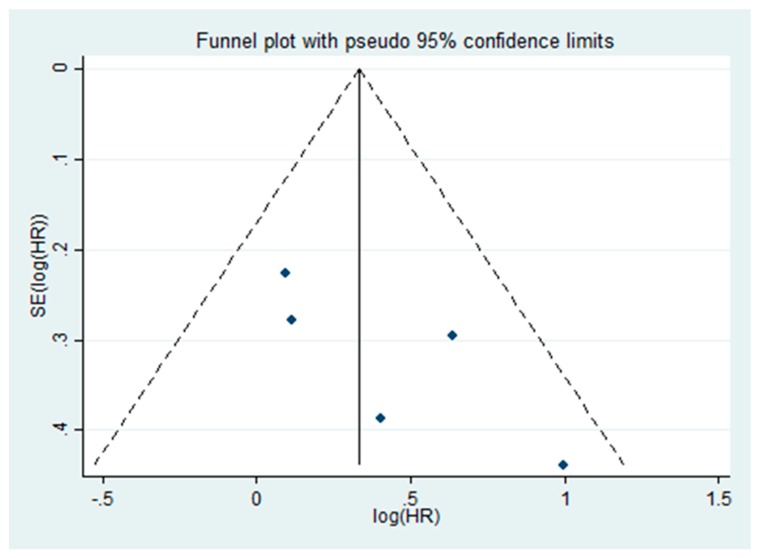
Funnel plot demonstrates no publication bias in five assessed studies in this meta-analysis.

The impact of focal NED in RP specimen on BCR using HR was investigated in five studies including a total of 944 patients [16,17,19,20,23]. All five studies used immunohistochemical CgA expression as NED marker. The Cochrane Q test (chi-square 3.24, *p* = 0.276) and the I2 test (I^2^ = 21.7%) revealed no heterogeneity. Therefore, we used a fixed-effect model. The forest plot (Figure 2) shows that focal NED was significantly associated with BCR after RP (pooled HR: 1.39, 95% CI 1.07‒1.81). Funnel plot analysis did not identify any publication bias (Figure 3).

## 3. Discussion

This systematic review and meta-analysis aimed to elucidate the prognostic value of focal NED in conventional PC on disease progression and survival after RP. We found that PC patients with focal NED in their RP specimen have an increased probability of BCR compared to patients without focal NED. However, this conclusion is based on mostly small retrospective studies making strong recommendations impossible. Moreover, the impact of focal NED in RP specimen on other endpoints such as cancer-specific and overall survival was only assessed in a limited number of studies, further weakening the possibility of making solid recommendations.

Neuroendocrine cells do not express prostate specific antigen (PSA) and androgen receptors [8,9]. Rapid disease progression in patients with a low serum PSA in pure neuroendocrine PC such as small cell carcinoma reflects the aggressive behavior of these tumors and the difference from standard follow-up strategies used for PC [26]. There is no clear understanding regarding the exact function of neuroendocrine cells in the prostate, but these cells are known to contribute to the inhibition of cellular apoptosis in PC through modulation of factors such as survivin, thereby enhancing the likelihood of BCR after RP [27,28,29]. Moreover, neuroendocrine cells may regulate the growth process of epithelial cells in prostate tissue by secretion of neuropeptides (e.g., bombesin, calcitonin, and serotonin), growth factors (e.g., vascular endothelial growth factor), and factors degrading the extracellular matrix (e.g., urokinase plasminogen activation system) [30,31,32,33].

Pathologic characteristics such as Gleason score and TNM classification of malignant tumors are well established prognostic factors after RP providing important information regarding the likelihood of BCR and survival [2]. NED formation in conventional PC treated with ADT is associated with rapidly progressive hormone resistant disease [34]. However, it still remains controversial whether patients with focal NED in conventional PC without history of ADT have worse prognosis when compared to those without focal NED. In our study, the number of patients included in studies reporting significant impact of NED on defined oncologic outcomes are comparable to those studies that showed no significant difference. One could conclude that there was no sample size effect on the difference of the results between studies. Currently, there is no recommendation for routine immunohistochemical staining of prostatic adenocarcinoma for neuroendocrine markers [35].

Neuroendocrine PC cells may not produce and/or leak PSA in the same amount as conventional PC. Therefore, monitoring with serum PSA evaluation is not ideal to identify progressive disease in patients harboring focal NED PC [36]. Due to the significant effect of focal NED on disease-specific and overall survival outcomes in some of the included studies in our review, serum neuroendocrine markers such as CgA and NSE might be considered as tumor markers for monitoring of PC patients who harbor focal NED on their RP [37]. This assumption needs, however, to be validated in well-designed studies.

Although the current study represents the first systematic review and meta-analysis demonstrating the prognostic impact of focal NED in conventional PC on significant oncologic outcomes including BCR after RP, it has some limitations. The retrospective nature of the studies, generally small cohorts, variability in CgA immunohistochemistry and scoring, variation in patients’ characteristics, and endpoint heterogeneity across studies limited the quality of the data and precluded further strong recommendations. Further prospective well designed studies considering other NED tissue markers such as NSE and synaptophysin might help clarify the prognostic value of NED in conventional PC.

## 4. Materials and Methods

### 4.1. Searching Strategy

Two independent reviewers conducted a full electronic literature search using PubMed, Scopus, Web of Science, and Cochrane Library on December 2018 to find relevant studies for this systematic review and meta-analysis according to the Preferred Reporting Items for Systematic Review and Meta-Analysis (PRISMA) guidelines. The search terms used were (“neuroendocrine differentiation” OR “neuroendocrine” OR “chromogranin A” OR “CgA” OR “neuron-specific enolase” OR “NSE” OR “serotonin” OR “Synaptophysin” OR “5-hydroxyindoleacetic acid OR 5-HIAA”) AND (“prostate” OR “prostatic” OR “prostate cancer” OR “radical prostatectomy”). Disease progression (including BCR, local recurrence, and distant metastasis) and survival data were our primary outcomes of interest.

### 4.2. Inclusion Criteria

The population, intervention, comparator, outcome, and study design (PICOS) approach was used to define the eligibility criteria: full text studies which assessed the association between focal NED in conventional prostate adenocarcinoma (population) in RP (intervention) specimen without history of neo-adjuvant therapy and post-operative prognosis included disease progression or survival were considered eligible. We excluded studies in languages other than English, review articles, meeting abstracts, case reports, replies, expert opinions, editorials, or commentaries. To perform meta-analysis, we included studies comparing positive RP specimen tissue staining for NED using predefined markers with patients without tissue staining for NED (comparator) to determine independent predictors of the mentioned oncological outcomes (outcome) after RP using multivariate Cox regression or logistic regression analysis (study design). 

### 4.3. Data Extraction

The full text of relevant studies were evaluated by two independent authors. In case of more than one study of the same cohort, we included only the largest or most recent study. Data were extracted on first author, year of publication, country of study, study design, recruitment period, total number of patients, NED tissue markers, oncological end outcomes, demographic and clinicopathological characteristics, and follow-up duration. Independent correlation of concomitant focal NED in prostate adenocarcinoma with oncologic outcomes were retrieved. 

### 4.4. Statistical Analyses and Bias Risk Assessment

We extracted reported HRs and 95% CIs to calculate cumulative effect size of studies which presented the association between focal NED of RP specimen as a prognostic factor and progression-free survival defined by BCR rate. Studies presenting HR using multivariate Cox proportional hazard regression model were included in meta-analysis. STATA/MPTM, version 14.2 (Stata-Corp, College Station, TX, USA) was used to perform meta-analysis. Heterogeneity between the studies included in the meta-analysis was assessed using Cochrane Q test and I^2^ statistics. An I^2^ > 50% and p-value < 0.05 in the Cochrane Q test implied that heterogeneity existed. With no heterogeneity among selected studies, we considered fixed effect models to calculate pooled HRs. Visual inspection of a funnel plot was carried out to identify publication bias in our meta-analysis. We used the ROBINS-I (“Risk Of Bias In Non-randomised Studies - of Interventions”) to assess the risk of bias in 16 included studies [38].

## 5. Conclusions

In this systematic review and meta-analysis, we detected a significant association of focal NED in conventional PC with oncologic outcomes including BCR after RP. Nevertheless, well-designed prospective studies overcoming inherent limitations of the current data are needed to confirm these findings. We suggest the assessment of focal NED in RP specimen, to prospectively assess the prognostic value in clinical decision making (i.e., ADT) and patient counselling.

## Figures and Tables

**Table 1:** Study characteristics of 16 studies assessing the role of neuroendocrine differentiation tissue markers in oncological outcomes after radical prostatectomy.

Author	Year	Region	Design	Recruitment Period	No. pts	Markers	Oncological End Point
Cohen [15]	1994	USA	Retrospective	1986–1989	38	CgA, NSE	Disease progression (LR, BCR, DM)
Noordzij [25]	1995	Netherlands	Retrospective	1977–1987	90	CgA	Disease progression (LR, DM), CSS
Bubendorf [14]	1996	Switzerland	Retrospective	1978–1993	137	CgA, NSE	Disease progression (LR, BCR, DM)
Weinstein [22]	1996	USA	Retrospective	N/A	104	CgA	PFS (BCR)
Theodorescu [24]	1997	USA	Retrospective	1970–1984	71	CgA	DSS, Long-term Survival
Abrahamsson [10]	1998	USA	Retrospective	1973–1989	87	S, CgA, NSE	Disease progression (LR, BCR, DM)
Krupski [18]	2000	USA	Retrospective	1970–1984	42	CgA	DSS (LR, BCR, DM)
Ahlgren [16]	2000	Sweden	Retrospective	N/A	53	CgA	PFS (BCR)
Bostwick [11]	2002	USA	Retrospective	1987-1992	196	S, CgA	DM, CSD, All cause death
Revelos [20]	2007	Greece	Retrospective	N/A	130	CgA	PFS (BCR)
Gunia [17]	2008	Germany	Retrospective	1996–2003	528	CgA	BFS (BCR)
Veltri [21]	2008	USA	Retrospective	1975–1991	105	CgA	PFS (LR, BCR, DM)
Ishida [12]	2008	Japan	Retrospective	N/A	RP (50) Vs NADT +RP (46)	S, CgA	BCR
Ma [19]	2010	Japan	Retrospective	N/A	RP (114) of PCa cases (435)	CgA	BFS (BCR)
Heinrich [13]	2011	Germany	Retrospective	N/A	175	S, CgA	BCR
Genitsch [23]	2017	Switzerland	Retrospective	1989–2006	119	CgA	PFS (BCR), CSS, OS

S: serotonin, CgA: chromogranin A, NSE: neuron-specific enolase, RP: radical prostatectomy, PCa: prostate cancer, N/A: not available, LR: local recurrence, BCR: biochemical recurrence, DM: distant metastasis, PFS: progression-free survival, CSD: cancer-specific death, BFS: biochemical free survival, CSS: cancer-specific survival, NADT: neo-adjuvant androgen deprivation therapy, OS: overall survival.

**Table 2:** Patient characteristics in 16 studies assessing the prognostic role of neuroendocrine differentiation after radical prostatectomy.

Author	Age, Year (mean/median)	Pre-Operative PSA, mg/dl (n)	Surgical GS (n)	Pathological Stage (*n*)	Follow-up Duration	Independent Correlation with Oncologic Outcomes
Cohen [15]	N/A	N/A	≤6 (20), 7 (14), ≥8 (4)	II† (22), III (16)	Mean: 50.5 months (range, 2–77)	NS
Noordzij [25]	62 (range, 47–74)	N/A	≤6 (26)/7 (36)/≥8 (28)	T2 (22), T3 (66), T4 (2), N+ (7)	Mean: 86 months (range, 1–203)	NS
Bubendorf [14]	65.3 (range, 45–82)	N/A	<7(68), ≥7 (69)	PT1 (4), PT2 (43), PT3 (90), N+(34)	Mean: 5.4 years (range, 1–15)	NS
Weinstein [22]	N/A	N/A	≤6 (59), >6 (45)	Organ confined (21%) SVI (0), LNI (0)	Mean: 8 years (range,7–10)	S
Theodorescu [24]	60.5 (range, 42–72)	N/A	≤7 (48), ≥8 (23)	Capsular penetration: −(37), +(31), N/A(3) SVI: −(50), +(13), N/A(8) LNI: −(13), +(1), N/A(57)	N/A	S
Abrahamsson [10]	66 (range, 50–77)	N/A	Mean GS: 6–7	A (1), B (27), C (50), D (9)	Mean: 4.2 years (range, 1.8–10.1)	NS
Krupski [18]	62 (range, 42–72)	N/A	≤6 (22), 7 (3), ≥8 (17)	N/A	Median: 10 years (range, 3.5–20)	NS
Ahlgren [16]	N/A	<10 (24), 11–20 (22), >20 (7)	≤6 (19), 7 (16), ≥8 (18)	T1b-T1c (22), T2-3 (31)	Mean: 39 ± 1 months	NS
Bostwick [11]	65.7 (range, 47–79)	Median: 21.4 (range, 0.9–616)	N/A	N+ (196)	Mean: 6.8 years (range, 0.3–11)	NS*
Revelos [20]	66 (range, 47–76)	Median: 9.23 (2.5–45.0)	≤6 (29), 7 (75), ≥8 (26)	ECE: +(70) −(60)/SVI: +(34) −(96), LNI: +(10) −(120)	Median: 28 months (1–97)	NS
Gunia [17]	63.8 (range, 44–79)	≤20 (472), >20 (56)	≤6 (316), 7 (157), ≥8 (55)	T2 (367), T3 (149), T4 (12), N0 (412), N1 (38), Nx (78)	Median: 46.4 months (range, 10–116)	S
Veltri [21]	59.62	N/A	< 7 (64), ≥ 7 (41)	T2: (75), >T2 (30) SVI: +(1), −(104) LNI (0)	Mean: 17.3 years (range:2–26)	NS
Ishida [12]	69 (range, 54–78)	Mean 7.5 (range 0.0–50.3)	RP: ≤6 (25), 7 (21), ≥8 (4)/NADT +RP: ≤6 (13), 7 (8), ≥8 (12)	I (24), II (29), III (25), IV(12)	N/A	NS
Ma [19]	70.28±7.43	N/A	≤6 (14), 7 (202), ≥8 (164), N/A (55)	T1a-bN0M0 (10), T1c-2N0M0 (191), T3-4N0M0 (83), T1-4N1M0-1 (25), T1-4N0-1M1 (126)	N/A	S
Heinrich [13]	63.3 ± 5.9 years	N/A	≤6 (86), 7 (63), ≥8 (24)	T2 (85), T3 (86), T4 (3)	Medium:17.1 months (range, 2–44)	NS
Genitsch [23]	65 (range, 45–75)	N/A	≤6 (12), 7 (63), ≥8 (44)	T2: 14, T3:105, N+ (119)	Median: 5.9 years (0.1–15.2)	NS

RP: radical prostatectomy, N/A: not available, NED: neuroendocrine differentiation, NADT: neo-adjuvant androgen deprivation therapy, PSA: prostate-specific antigen, S: significant, NS: not-significant, ECE: extra capsular extension, SV: seminal vesicle invasion, LNI: lymph node invasion. *serotonin in benign epithelium was associated with cancer specific death but not distant metastasis or all cause survival. † Stage II: organ confined disease, III: extra capsular extension, seminal vesicle invasion, positive surgical margin.

**Table 3:** Risk of bias assessment for individual studies using the “Risk Of Bias In Non-randomised Studies - of Interventions” tool (ROBINS-I).

Author	Confounding	Participant Selection	Classification of Interventions	Deviations from Intended Intervention	Missing Data	Measurement of Outcomes	Selection of the Reported Result	Overall
Abrahamsson [10]	Serious	Serious	Low	Serious	Low	Moderate	Low	Serious
Ahlgren [16]	Serious	Serious	Low	Moderate	Low	Low	Low	Serious
Bostwick [11]	Serious	Serious	Low	Serious	Low	Moderate	Moderate	Serious
Bubendorf [14]	Serious	Serious	Low	Moderate	Low	Low	Moderate	Serious
Cohen [15]	Serious	Serious	Low	Serious	Low	Moderate	Low	Serious
Gunia [17]	Serious	Serious	Low	Serious	Low	Moderate	Low	Serious
Heinrich [13]	Serious	Serious	Low	Serious	Low	Low	Moderate	Serious
Ishida [12]	Serious	Serious	Low	Serious	Low	Moderate	Moderate	Serious
Krupski [18]	Serious	Serious	Low	Serious	Low	Moderate	Moderate	Moderate
Ma [19]	Serious	Serious	Low	Low	Low	Moderate	Low	Serious
Noordzij [25]	Serious	Serious	Low	Moderate	Low	Low	Moderate	Serious
Revelos [20]	Moderate	Serious	Low	Serious	Low	Low	Moderate	Serious
Theodorescu [24]	Serious	Moderate	Low	Serious	Low	Moderate	Low	Serious
Veltri [21]	Serious	Serious	Low	Serious	Low	Low	Moderate	Serious
Weinstein [22]	Moderate	Moderate	Low	Moderate	Low	Moderate	Low	Moderate
Genitsch [23]	Serious	Serious	Low	Serious	Low	Moderate	Moderate	Serious

## Abbreviations

NED	neuroendocrine differentiation
PC	prostate adenocarcinoma
RP	radical prostatectomy
BCR	biochemical recurrence
PRISMA	Preferred Reporting Items for Systematic Review and Meta-Analysis
CgA	chromogranin A
NSE	neuron specific enolase
PSA	prostate specific antigen
ADT	androgen deprivation therapy
